# F4. LINKING LIFE EVENTS WITH NEGATIVE AFFECT AND PSYCHOTIC EXPERIENCES IN DAILY LIVES OF YOUTH: STRESS SENSITIVITY AS A PUTATIVE MECHANISM?

**DOI:** 10.1093/schbul/sby017.535

**Published:** 2018-04-01

**Authors:** Christian Rauschenberg, Jim van Os, Dimitri Cremers, Matthieu Goedhart, Jan Schieveld, Ulrich Reininghaus

**Affiliations:** 1 Maastricht University; 2 University Medical Center Utrecht; 3 Tilburg University; 4 Maastricht University Medical Center

## Abstract

**Background:**

Negative life events are associated with a range of mental disorders, including psychosis. However, evidence on underlying mechanisms remain scarce. The current study aimed to investigate whether life events (e.g. intrusive threat, experience of loss, illness) impact on the sensitivity towards stress in daily lives of youth.

**Methods:**

The Experience Sampling Method was used to measure momentary stress (i.e. event-related, activity-related, social), negative affect, and psychotic experiences in a sample of 42 help-seeking adolescents and young adults (service user), 17 siblings, and 40 comparison subjects (controls). Life events during lifetime and the previous year as well as depressive, anxiety, and psychotic symptoms were assessed.

**Results:**

Stress sensitivity, that is, the associations between momentary stress and (i) negative affect and (ii) psychotic experiences, was modified by lifetime and previous negative life events in service users. While there was strong evidence for increased negative affect and psychotic experiences in service users when high vs. low levels of lifetime exposure to negative life events were compared a pattern of resilience was evident in controls with no marked differences in the magnitude of associations comparing high vs. low exposure levels. However, in controls, exposure to life events during the previous year were also found to impact on the stress sensitivity in daily life. Less consistent findings were observed in siblings.

**Discussion:**

Our findings point to the importance of time that has passed between exposure to and impact of life events on stress sensitivity: while the detrimental effects may attenuate in controls over time, service users appeared to be at greater risk of negative long-term effects. Thus, stress sensitivity may constitute an important risk and resilience mechanism through which adverse life events impact on mental health in youth. Targeting stress sensitivity in daily life through ecological momentary interventions, potentially with stronger effects shortly after stress exposure, may represent a promising novel therapeutic approach.

